# Evolving microbiology laboratories: mastering automated culture-based processes and molecular assays, an institutional experience

**DOI:** 10.3389/fcimb.2026.1766429

**Published:** 2026-04-20

**Authors:** Abdessalam Cherkaoui, Gesuele Renzi, Adrien Fischer, Mireille Tittel-Elmer, Mikaël Tognon, Patrice François, Vladimir Lazarevic, Jacques Schrenzel

**Affiliations:** 1 Bacteriology Laboratory, Division of Laboratory Medicine, Department of Diagnostics, Geneva University Hospitals, Geneva, Switzerland; 2 Division of General Internal Medicine, Department of Medicine, Geneva University Hospitals, Geneva, Switzerland; 3 Genomic Research Laboratory, Department of Molecular Microbiology, Faculty of Medicine, Geneva, Switzerland

**Keywords:** antimicrobial susceptibility testing, artificial intelligence, genotypic assay, gram staining, phenotypic assay, sequencing, total laboratory automation

## Abstract

Total laboratory automation (TLA) in microbiology integrates robotic specimen processing, automated conveyor systems, smart incubators, and high-resolution digital imaging to automate culture-based workflows from specimen setup to plate reading. Successful implementation requires careful planning, including assessment of existing laboratory infrastructure and a strategy for interfacing third-party instruments and information systems. Major barriers include capital investment, interoperability, and the need for standardized information technology interfaces. Recent advances in artificial intelligence (AI), particularly machine learning and convolutional neural networks, have extended the value of TLA by enabling automated image interpretation, culture plate screening, and predictive analyses. These tools can reduce manual workload and turnaround time while improving standardization. In this review, drawing primarily on our institutional experience, we examine the impact of TLA and AI on diagnostic microbiology workflows, implementation strategies, and performance assessment. We also discuss automated digital microscopy, the integration of phenotypic and molecular methods, and the principal limitations that still constrain broader adoption. Finally, we highlight the need for molecular diagnostic stewardship to preserve clinical relevance and cost-effectiveness.

## Introduction

Clinical microbiology laboratories are being reshaped by the convergence of total laboratory automation (TLA), digital imaging, and artificial intelligence (AI) (Mencacci et al., 2023; Shelke et al., 2023). Together, these technologies support continuous, high-throughput processing of clinical specimens while improving standardization, traceability, and turnaround time (Cherkaoui and Schrenzel, 2022; Dien Bard et al., 2025; Graf et al., 2025). Their adoption has been driven by growing specimen volumes, pressure on staffing, and the need to control operating costs without compromising analytical quality (Culbreath et al., 2021; Trigueiro et al., 2024). Four components are central to this transformation: robotic systems for specimen handling, a robust information technology infrastructure, affordable AI models that support timely interpretation of digital data, and effective integration of culture-based and molecular assays. In this review, we focus on how these elements interact in practice, what they improve reliably, and where important limitations remain.

## Automated microscopy digital imaging coupled to deep-learning-based image analysis module

Until recently, Gram stain remained central to presumptive etiologic diagnosis and empirical treatment decisions. However, its performance is operator dependent and varies according to specimen type and specimen quality (Samuel et al., 2016a, b). Manual Gram stain microscopy is also labor-intensive and time-consuming. To address these limitations, several groups have evaluated automated digital microscopy combined with deep-learning-based image analysis. Walter et al. (2024) assessed a fully automated digital microscope with an AI-based application for Gram stain interpretation from positive blood cultures. Positive and negative percent agreement with conventional microscopy reached 95.8% and 98.0% for Gram-positive cocci in clusters, 87.6% and 99.3% for Gram-positive cocci in pairs and chains, 97.4% and 97.8% for bacilli, and 83.3% and 99.3% for yeasts. The reported limit of detection was 10^5^ CFU/mL. In a comparable study, Smith et al. (2018) used a deep convolutional neural network for automated blood-culture Gram stain interpretation and reported sensitivities of 98.4%, 93.2%, and 96.3% for Gram-positive cocci in chains/pairs, Gram-positive cocci in clusters, and Gram-negative rods, respectively, with specificities of 75.0%, 97.2%, and 98.1%.

Despite these advances, the clinical value of Gram stain is now less consistent across specimen types. Ngo Nsoga et al. (2023) showed that Gram stain was no longer necessary for cerebrospinal fluid diagnostics when modified Reller criteria were applied. In our institution, a retrospective comparison of Gram stain with culture for pleural fluid showed limited performance, with a sensitivity of 64% and a specificity of 50% (n = 65; unpublished data). These observations suggest that automation alone does not justify maintaining Gram stain in all workflows; its role should be reassessed according to specimen type, pretest probability, and the availability of faster molecular alternatives.

Automated microscopy coupled to a deep learning model was evaluated for fungal detection in 236 skin samples, 50 nail samples, and 6 hair samples. The results were compared to the manual approach. The sensitivity and specificity for fungal detection were 99.5% and 91.4% for the skin samples, 95.2% and 100% for the nail samples, and 60% and 100% for the hair samples (Gao et al., 2021). Automated acid-fast bacilli (AFB) smear microscopy scanning coupled to deep-learning-based image analysis was assessed in a retrospective study conducted by Desruisseaux et al (Desruisseaux et al., 2024). A total of 286 non-consecutive auramine-O-stained respiratory and pleural samples slides were compared to the manual method. Mycobacterial culture positivity rate was 24.1%. Compared to the mycobacterial cultures, the sensitivity and the specificity of the assisted digital microscopy were 90.7% (95%CI, 81.7%–96.2%) and 91.9% (95%CI, 87.4%–95.2%), respectively. Whereas the proof of principle appears promising, automated microscopy digital imaging assisted by deep-learning-based image analysis still requires further investigations to improve its efficiency and determine its real value within the modern microbiology laboratory workflows.

As automation and AI become more integrated into microbiology laboratories, the place of Gram stain warrants re-evaluation. In highly automated settings with high-throughput, end-to-end workflows, Gram stain may be best reserved for selected specimen types in which it adds immediate clinical value. By contrast, it remains indispensable in small-to-medium-sized laboratories and in settings where automated or molecular alternatives are unavailable.

## Culture-based process: total automation and digital imaging

The core functions of a clinical microbiology laboratory are to detect and identify pathogens, determine antimicrobial susceptibility, and support infection prevention. Automation has substantially improved how these tasks are performed. Robotic specimen processing, automated incubation with time-lapse imaging, digital plate reading, and automated antimicrobial susceptibility testing (AST) can improve consistency, traceability, and turnaround time while reducing repetitive manual work. Reported economic benefits include higher productivity, better allocation of skilled personnel, optimized consumable use, improved reproducibility, lower error rates, and faster reporting (Culbreath et al., 2021; Trigueiro et al., 2024). However, the scale and nature of these benefits depend strongly on local organization and policy, and should not be generalized without context.

Economic objectives differ across laboratories and health-care systems. In some settings, gains from automation are measured primarily as reductions in cost per specimen or increases in throughput. In our institution, these gains were used instead to develop a more versatile and multi-skilled workforce, improve the working environment, increase scheduling flexibility, and support innovation. This distinction is important because the value of TLA should be assessed not only by productivity metrics, but also by how effectively it strengthens laboratory resilience and quality.

Several retrospective observational studies, comparing pre- and post-TLA periods have shown the positive impact of TLA on the reduction of TATs. For urine samples, the TAT for negative reports decreased by almost half from 52.1 h to 28.3 h (p <0.001) (Cherkaoui et al., 2020); from 73.7h (IQR: 35.6-50.7) to 40.0h (IQR: 35.6-50.7) (Trigueiro et al., 2024); from 24.3h pre-TLA to 23.0h post-TLA (p <0.001) (Kritikos et al., 2024); from 52.1h to 49.6h (P<0.001) (Zhang et al., 2021). Other specimen types have also benefited from the reduction of TAT: from 70.3h (IQR: 63.5-93.1) to 48.2h (IQR: 44.8-67.7) for vaginal swabs (Trigueiro et al., 2024); from 97h to 53.5h (Δ43.5h) for blood cultures and from 73h to 58h (Δ20h) for biological fluid samples (Fontana et al., 2023); from 64.4h to 61.4h (P<0.001) for sputum, from 68.5h to 66.6h (P<0.001) for blood cultures, from 86.8h to 64.3h (P = 0.007) for cerebrospinal fluid samples (Zhang et al., 2021), and from 50.7h to 26.3h (p<0.001) for MRSA screening specimens, from 50.2 h to 43.0h (p<0.001) for ESBL screening specimens, and from 50.6 h to 45.7h (p<0.001) for VRE screening specimens (Cherkaoui et al., 2020).

A major next step in automation is the use of computer vision for digital culture plate reading. When combined with chromogenic media, AI-assisted image analysis can achieve performances comparable to nucleic acid amplification tests for selected applications, such as the detection of *Streptococcus agalactiae* (Baker et al., 2020), and may enable simpler and less costly workflows. For urine cultures, AI-assisted plate reading has shown greater than 90% agreement with manual interpretation on chromogenic agar (Dauwalder et al., 2021). Colony segregation software has also performed well on standard media: Faron et al. reported a sensitivity of 99.8% and a specificity of 72.0% for growth versus no-growth discrimination in urine cultures (Faron et al., 2020). In a broader study including 3, 844 plates from multiple specimen types, Jacot et al. reported a sensitivity of 99.1% and a specificity of 93.4% after 24 h of incubation (Jacot et al., 2024). These data support routine use for triage and negative screening, although performance still depends on culture media, specimen types, and validation strategy.

Computer vision software now streamlines culture-based workflows. For example, PhenoMATRIX^®^ PLUS (Copan, Brescia, Italy) can sort and process culture plates according to predefined rules, automatically releasing negative results to the electronic patient records and discharging corresponding negative plates from incubators. In a previous study, we assessed the performance of PhenoMATRIX^®^ and PhenoMATRIX^®^ PLUS coupled to chromogenic agar plates for methicillin-resistant *Staphylococcus aureus* (MRSA) screening from nasal and inguinal/perineal swabs (Cherkaoui et al., 2024). Compared with the manual workflow, PhenoMATRIX^®^ achieved 99.8% (95% CI, 99.2%-99.9%) sensitivity and 99.1% (95% CI, 99.0%-99.2%) specificity. PhenoMATRIX^®^ PLUS showed 100% (95% CI, 92.1%-100%) sensitivity and 95.2% (95% CI, 93.8%-96.1%) specificity. Anterior nares specimens (n=1, 593) from three sites, two in the United States and one in Canada, were analyzed by the BD Kiestra TLA system combined with an imaging application that uses AI to identify MRSA specific colonies on CHROMagar chromogenic media. Compared to the manual reading, the sensitivity was 98.2% (95% CI, 96.0%-99.3%) and the specificity was 96.7% (95% CI, 95.6% -97.6%) (McElvania et al., 2024).

**Figure 1:**
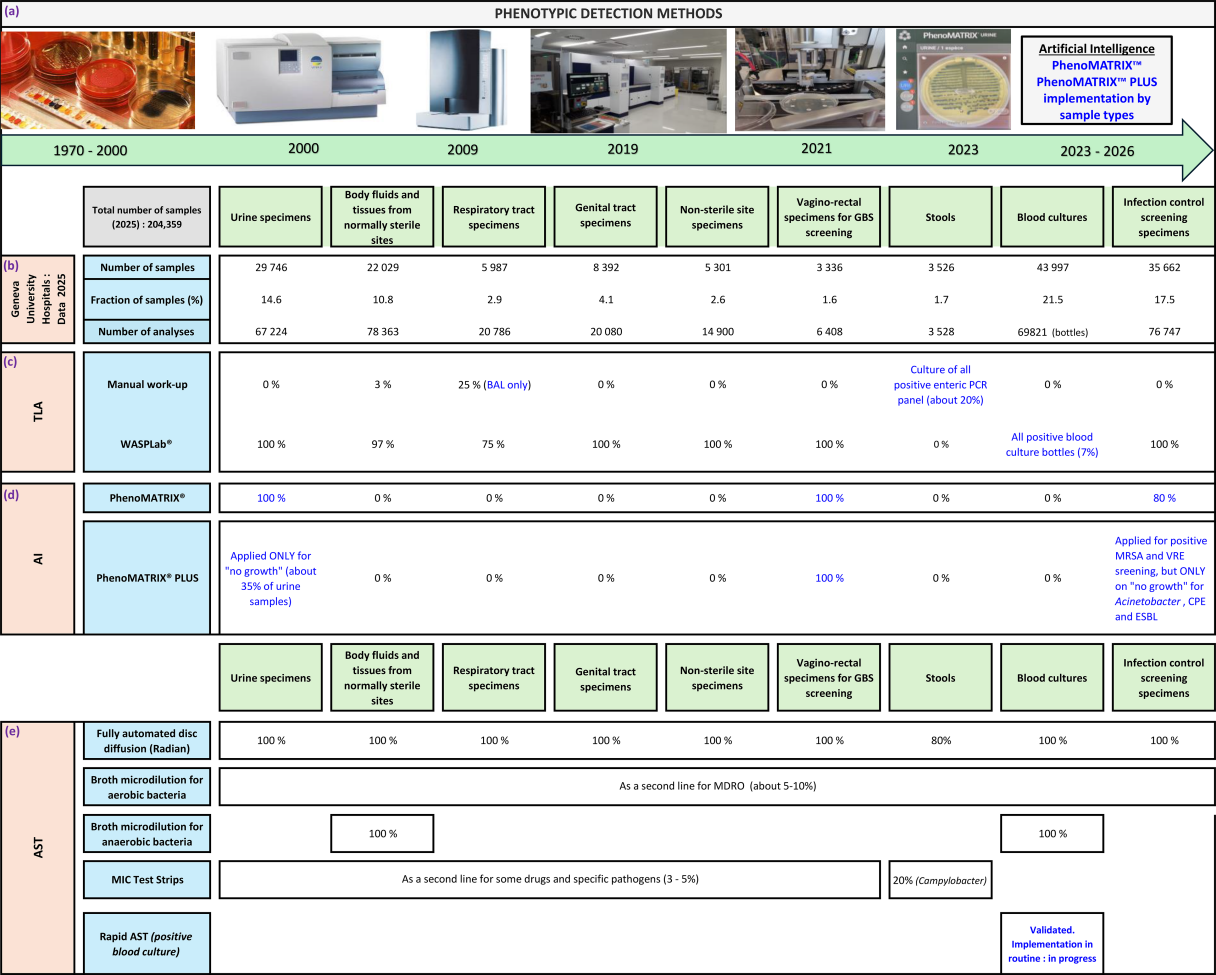
Key phenotypic methods and algorithms applied in bacteriology lab at Geneva university hospitals according to the specimen type and the clinical features. Clinical samples for mycobacteria analysis were not included. **(a)** evolution of phenotypic detection methods used in our laboratory, **(b–d)** total lab automation workflows, **(e)** antimicrobial susceptibility testing workflows.

Automated culture reading has also been evaluated for additional pathogens and specimen types (Cherkaoui et al., 2024; 2025a, b). In our laboratory, these tools have improved workflow control and resource allocation. **Figure 1a** summarizes the evolution of phenotypic detection methods used in our laboratory since the 1970s. MALDI-TOF MS marked an important transition, but the most substantial operational gains were achieved after implementation of TLA with AI-assisted image analysis. At present, 97% of specimen types considered amenable to automation are processed through TLA in our laboratory. The remaining 3% still require manual preparation or inoculation before incubation on the WASPLab, including catheters, vascular or orthopedic prostheses, and surgical devices (**Figures 1b–d**). Further gains are likely as AI applications are extended beyond chromogenic media, but their generalizability will require careful validation.

## Automated antimicrobial susceptibility testing

Since the earliest AST studies, laboratories have sought methods that improve standardization while accelerating treatment guidance (Khan et al., 2019). In a previous study, we compared the Vitek 2 system with fully automated disk diffusion susceptibility testing using the Colibri coupled to the Radian under routine conditions (Cherkaoui et al., 2021). Overall categorical agreement was 99.3% for Enterobacterales, 98.6% for *Pseudomonas aeruginosa*, 99.4% for *Staphylococcus* spp., and 99.4% for *Enterococcus* spp. The very major errors observed for *P. aeruginosa* with Vitek 2 were linked to heteroresistant populations. In our laboratory, fully automated disk diffusion now serves as the first-line AST method for approximately 98% of panels, with broth microdilution and MIC test strips reserved for selected organisms and complex resistance profiles (**Figure 1e**). Comparable results have been reported by others using similar workflows (Vanstokstraeten et al., 2025), supporting the robustness of this approach in routine practice.

A fully automated EUCAST rapid AST (RAST) directly from positive blood cultures was evaluated in a previous study focusing on three different aspects: i) determination of RAST performances compared to standard EUCAST disk diffusion testing; ii) detection of resistance mechanisms and specific resistance patterns by RAST; and iii) evaluation of the unreadable inhibition zones and areas of technical uncertainties (ATU) at different incubation times against standard AST (Cherkaoui et al., 2022).

The key conclusions of this study were: i) trends observed in the phase-1 (spiked blood cultures) were confirmed in the clinical trial; ii) no discordant results at the categorical level were observed among the blood cultures of the same patient; iii) full automation of RAST decreases the percentages of ATU and the unreadable inhibition zones significantly, as compared to previous studies; iv) very major errors identified for *P. aeruginosa* were linked to hetero-resistance profiles (colonies growing in the inhibition halo of the standard method); v) double disk synergy testing enables accurate identification of ESBL by RAST at 6h; and vi) accurate detection of CPE was achieved using the RAST screening cutoff values.

Non-automated EUCAST RAST directly from positive blood cultures was compared to the BD Phoenix™ system using 354 positive blood cultures including 51 monomicrobial Gram-negative ESKAPEEc isolates (Cannella et al., 2025). In this study EUCAST RAST was performed manually. The authors observed that the performance of the non-automated EUCAST RAST differed widely depending on the bacterial species and the antibiotic tested.

Other research has assessed different rapid susceptibility testing approaches directly from positive blood cultures. For example, the automated system Alfred^60^AST was assessed on 92 Gram-negative and 84 Gram-positive bacteria. Compared to Vitek2, categorical agreement was 94.5% after discrepancy analysis (Mantzana et al., 2021). A further example, the Selux AST system coupled with positive blood culture (PBC) Separator was evaluated in a multicenter clinical trial for RAST directly from PBC (Streva et al., 2025). This technology applied is based on complementary viability and surface area fluorescence assays. Compared with the reference broth microdilution, the essential agreement was >95%. In a recent study, VITEK REVEAL was evaluated against Accelerate Pheno for AST directly from 128 Gram-negative BCs. Categorical and essential agreements were 94.3% and 96.0%, respectively (very major errors, 7.5%; major errors, 0.5%, and minor errors, 4.1%) (Simmons-Williams et al., 2026). Other reports showed comparable performances for the VITEK REVEAL (Bui et al., 2025; Park et al., 2026).

Because several approaches are now available for AST directly from positive blood cultures, method selection should be guided by laboratory needs rather than by analytical performance alone. Relevant factors include testing cost, degree of automation, positive blood-culture volume, staffing model, and compatibility with existing workflows. Reported accuracy is often high, but species-antibiotic combinations, resistance mechanisms, and local implementation constraints still influence real-world performance.

## Lab organization for the molecular assays

Low-multiplex molecular assays have been implemented on commercially available platforms according to analytical volume, turnaround time, assay performance, and cost. In our laboratory, the highest-volume assays, such as *Chlamydia trachomatis* and *Neisseria gonorrhoeae* detection, are performed on a high-throughput platform (Cobas 6800; Roche). Lower-volume assays and limited multiplex panels, such as MRSA and *Clostridioides difficile* testing, are run on systems that do not require batching, including BD Max (Becton Dickinson) and BioFire (bioMérieux). Only a limited number of assays are maintained on dedicated qPCR platforms because of low ordering frequency, as illustrated by our *Kingella kingae* assay (Cherkaoui et al., 2009). This organization reflects a pragmatic strategy that aligns platform complexity with clinical demand.

Further analysis of strains is often required for the characterization of specific antimicrobial-resistance determinants, such as carbapenemase-encoding genes (Haldorsen et al., 2021) or for detailed molecular typing (e.g. MRSA typing (Megevand et al., 2010). Rapid and cost-effective methods are also essential to investigate clonal transmission in nosocomial outbreaks. We have developed assays largely based on Multiple-Locus Variable-Number Tandem-Repeat Analysis (MLVA), i.e. the detection of low complexity repeats in the genome that are more susceptible to INDEL mutations and therefore constitute ideal gene targets for rapidly ruling out clonality. The method used for shallow genotyping MRSA is detailed in a previous study (Kuhn et al., 2010) and was further developed and adapted to tackle most of the relevant organisms causing nosocomial outbreaks such as the ESKAPE organisms (unpublished data). This shallow genotyping approach permits rapid turn-around time (a few hours) at an affordable cost. As part of routine surveillance at the Geneva University Hospitals, suspected outbreak events are now investigated using internally developed MLVA assays targeting the following bacterial pathogens: all ESKAPE organisms, *Serratia marcescens*, *Citrobacter freundii*, *Escherichia coli*, and *Neisseria meningitidis* among Gram-negative bacteria; and *Staphylococcus epidermidis*, *Enterococcus faecalis*, *Streptococcus pyogenes*, and *Clostridioides difficile* among Gram-positive bacteria.

Next-generation sequencing (NGS)-based approaches are progressively entering routine infectious disease diagnostics. Whole-genome sequencing of cultured isolates now complements conventional methods by providing high-resolution strain characterization, supporting investigation of suspected transmission events, and resolving taxonomic or serotyping uncertainties. However, its value is clearest when a specific epidemiologic or diagnostic question justifies the additional cost and analytical complexity.

**Figure 2:**
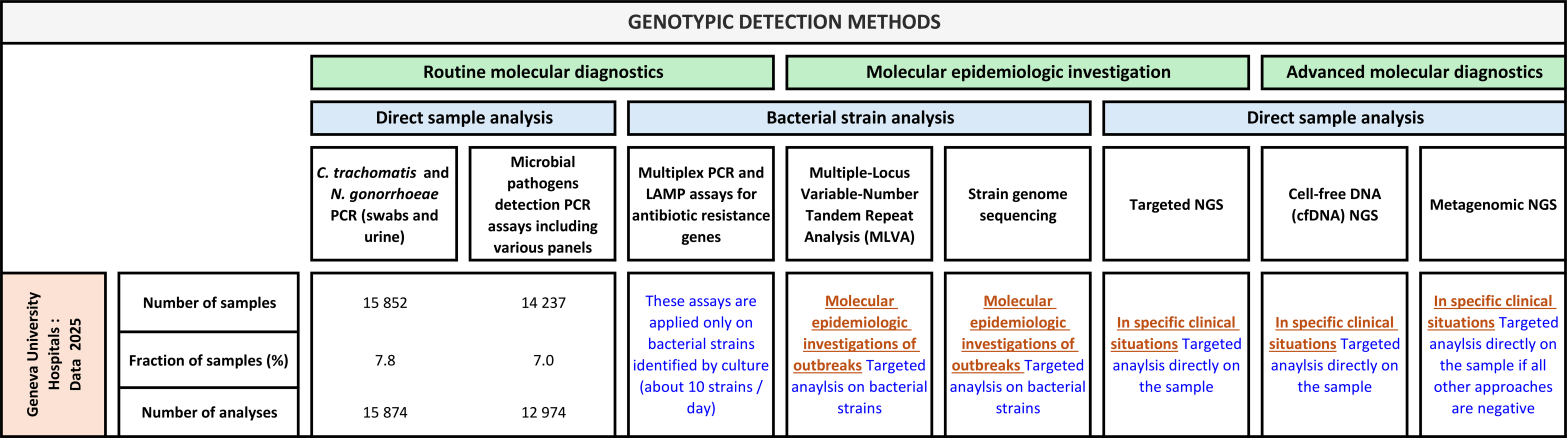
Key genotypic methods and algorithms applied in our institution according to the specimen type and the clinical features.

Targeted NGS approaches based on amplification or capture of conserved regions such as 16S, 18S, or internal transcribed spacer sequences enable simultaneous detection of multiple pathogens in mono- or polymicrobial samples and may improve sensitivity (Breitwieser et al., 2018). Metagenomic NGS (mNGS) takes an untargeted approach by sequencing all nucleic acids in a specimen and can identify unexpected, non-cultivable, or rare microorganisms while also detecting resistance or virulence genes (Chiu and Miller, 2019; Rodino and Simner, 2024). Plasma cell-free DNA NGS provides noninvasive detection of large pathogen panels within 24 to 48 h using commercial assays (Blauwkamp et al., 2019; Jung et al., 2025; Kim et al., 2025; Sim et al., 2025). Despite these advantages, these methods remain costly, require specialized wet-lab and bioinformatics expertise, and often generate findings that need multidisciplinary interpretation. Their current role is therefore selective rather than routine, and in our institution they are reviewed through dedicated stewardship pathways (**Figure 2**).

## Integration of phenotypic and molecular approaches

From a laboratory management perspective, automated phenotypic assays generally remain the preferred first-line strategy because they combine short turnaround time, scalability, moderate cost, and straightforward integration into laboratory information systems. This makes a funnel-based diagnostic model attractive: broad, automated phenotypic screening is performed first, and more expensive molecular assays are reserved for selected positive or unresolved cases. This model is operationally efficient, but it should not be applied uncritically because some clinical contexts favor molecular testing upfront.

**Figure 3:**
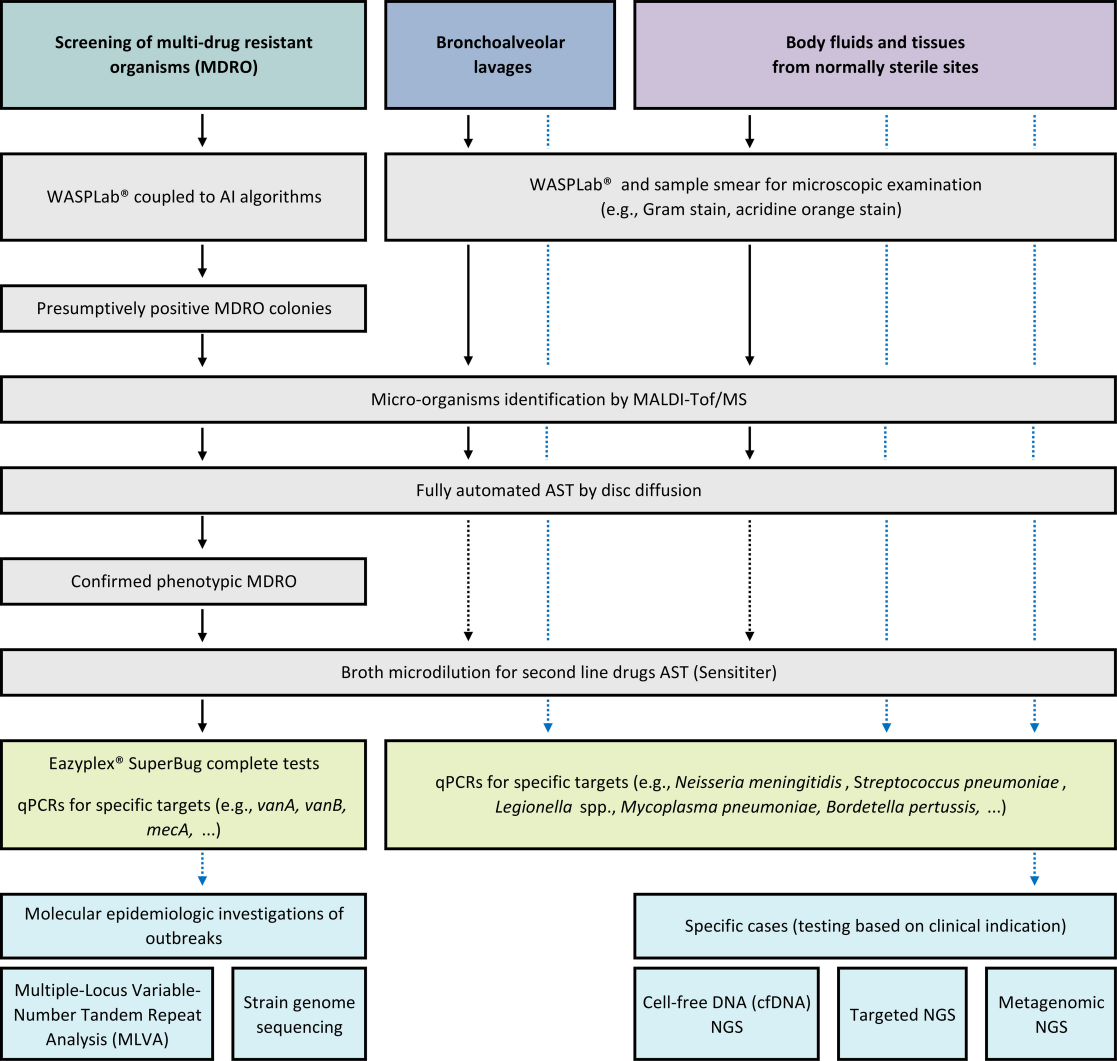
Illustration of comprehensive workflows for bacterial samples analysis. The workflows detailed in this figure consider the specific characteristics of our institution and may differ across laboratories in other settings. Plain lines: routine workflows. Dotted lines: as applied for specific cases.

Two notable exceptions to this paradigm merit consideration. The first concerns stool screening, where the high analytical sensitivity of targeted molecular panels allows same-day exclusion of some enteric pathogens at an affordable cost, given the limited complexity of these assays. In this context, the diagnostic workflow is effectively reversed: molecular testing is performed upfront, and positive results subsequently prompt targeted culture on selective media (**Figure 3**).

The second exception relates to tuberculosis diagnosis, for which nested quantitative PCR assays have consistently demonstrated superior analytical sensitivity compared with phenotypic methods. Nonetheless, mycobacterial cultures performed in parallel remain essential to assess strain viability, perform phenotypic AST, and enable genotyping when required.

Phenotypic and PCR-based diagnostics should therefore be viewed as complementary rather than competing approaches. Their relative value depends on specimen type, likelihood of infection, prior antimicrobial exposure, and the underlying clinical question. At present, NGS-based methods remain second- or third-line tools in most workflows because their added sensitivity is offset by higher cost, greater interpretative complexity, and limited actionability in low-pretest-probability settings (**Figure 3**).

## Principles and implementation of molecular diagnostic stewardship

Molecular diagnostic stewardship requires several coordinated measures: clinician education on the indications, strengths, and limitations of available assays; close collaboration with infection control and infectious diseases teams; and controlled access to high-complexity methods such as plasma cell-free DNA NGS and mNGS on the basis of a clearly defined clinical question. This framework is necessary because faster or broader molecular detection is not inherently better; the clinical value of a result depends on context, actionability, and the likelihood that it changes patient management.

In infection control surveillance, phenotypic methods are typically favored as first-line tools because they support high sample throughput and rapid turnaround times. When an outbreak is suspected, rapid, low-resolution genotyping is performed to provide same-day actionable decision. Whole-genome sequencing is reserved for situations requiring higher molecular resolution, such as the investigation of unexpected transmission links or prolonged outbreaks, or research-driven objectives.

Although molecular assays can provide earlier or broader pathogen detection in selected settings, such as prior antibiotic exposure, culture-negative infection, or critical illness, indiscriminate use in patients with a low probability of infection is unlikely to improve outcomes and may increase cost, false-positive interpretation, and downstream testing. Stewardship is therefore not only a cost-containment strategy, but also a means of preserving diagnostic specificity and clinical relevance.

## Challenges in molecular diagnostics

Molecular diagnostics, particularly NGS-based methods, introduce substantial interpretative challenges. Their high analytical sensitivity increases the likelihood of detecting incidental organisms, asymptomatic carriage, and clinically irrelevant microorganisms, especially in polymicrobial specimens. Distinguishing colonization from infection, interpreting low-abundance signals, and accounting for environmental, reagent, or carryover contamination remain difficult, particularly in low-biomass samples. As a result, analytical sensitivity does not translate directly into clinical utility.

In such low-burden contexts, mNGS faces an additional limitation: the predominance of host-derived nucleic acids may substantially reduce microbial read recovery and complicate signal interpretation. Moreover, molecular methods detect nucleic acids rather than viable organisms, and persistent positivity may therefore occur despite adequate therapy. Although quantitative metrics are increasingly reported in NGS-based workflows, validated thresholds defining clinical relevance are lacking. Consequently, molecular findings must be integrated with clinical, microbiological, and epidemiological data.

## Discussion

Consensus guidance for TLA in microbiology emphasizes the transition from batch-based workflows to continuous, traceable, and increasingly paperless systems (Bourbeau and Ledeboer, 2013; Burckhardt, 2018). Core elements include automated inoculation, continuous incubation with digital image capture at predefined time points, integration of colony picking, MALDI-TOF MS identification and AST, and AI-assisted plate interpretation within systems such as BD Kiestra or Copan WASPLab (Burckhardt, 2018; Cherkaoui and Schrenzel, 2022; Trigueiro et al., 2024). However, implementation remains laboratory specific. Our review is based primarily on institutional experience and on studies used to validate these approaches locally. The workflows shown in the figures therefore illustrate one operational model rather than a universally applicable template. **Table 1** summarizes the metrics used to compare our laboratory with other centers.

**Table 1:** Metrics used to compare our laboratory with other centers.

Criteria	Metrics evaluated	Situation of our lab
System flexibility and modularity	Ability to adapt to different laboratory layouts and scale	System-1 is composed of 2 WASP, 2 "CO2 (5%) atmosphere" and 1 "air atmosphere" incubators.
Physical footprint and scalability	Space requirements, modularity for future expansion, and ease of implementing in existing lab layouts	The possibilities are rather limited by the avalaible lab surface area.
Workflow optimization	Impact on reduction of repetitive, manual tasks to enhance productivity	At present, 97% of specimen types considered amenable to automation are processed through TLA in our laboratory. The remaining 3% still require manual preparation or inoculation before incubation on the WASPLab, including catheters, vascular or orthopedic prostheses, and surgical devices.
IT and LIS connectivity	Ability to integrate seamlessly with existing Laboratory Information Systems (LIS)	Several months of development and testing were necessary. We are pressing ahead with our strategic development using an in-house LIS system.
Automated microscopy digital imaging coupled to deep-learning- based image analysis module	Performances and implementation	We evaluated an automated digital imaging of Gram-stained slides with on-screen reading against manual microscopy. The system was not implemtented in the routine.
Imaging and digital culture interpretation	High-resolution digital imaging capabilities at regular intervals	Automation significantly reduces incubation times and allows earlier culture readings for all the specimens types processed by TLA.
AI and software Integration	Use of algorithms to assist in reading plates	PhenoMATRIX and PhenoMATRIX PLUS were implemented for various specimen types.
Automated antimicrobial susceptibility testing (AST)	Automated and standardized AST	Fully automated antimicrobial disk diffusion susceptibility testing. System-2, solely dedicated to AST, is composed of 1 WASP, 1 Radian, and 1/2 "air atmosphere" incubator.
Workflow Efficiency (TAT)	Impact on reducing TAT from inoculation to actionable results	The median TAT for negative reports decreased for urine from 52.1h to 28.3h (p<0.001), for MRSA screening specimens from 50.7h to 26.3h (p<0.001), for ESBL screening specimens from 50.2h to 43.0h (p<0.001), and for VRE screening specimens from 50.6h to 45.7h (p<0.001).
Cost and labor impact	Potential reductions in full-time equivalents (FTEs) by decreasing manual plate handling	In our institution, these gains were used to develop a more versatile and multi- skilled workforce, improve the working environment, increase scheduling flexibility, and support innovation.

Implementation of TLA requires major upfront investment, staff training, adaptation of laboratory space, and substantial organizational change (Dien Bard et al., 2025). Reported performance metrics also remain difficult to compare across studies because of heterogeneity in culture media, incubation conditions, image interpretation rules, specimen handling, and specimen types (Lippi and Da Rin, 2019; Dauwalder et al., 2021; Jiang et al., 2022). Biological variation adds another layer of complexity, particularly for fastidious organisms and low-burden samples (Burckhardt, 2018; Cherkaoui et al., 2020; Antonios et al., 2021). As a result, published gains in turnaround time or accuracy should be interpreted cautiously unless validation conditions are clearly described.

TLA improves standardization and turnaround time for high-volume workflows, but important limitations remain (Lippi and Da Rin, 2019; Nam and Park, 2025; Plebani, 2026). These include high capital costs, dependence on specialized consumables, the need for dedicated or redesigned space, vulnerability to system downtime, limited handling of unusual or fastidious organisms, staff retraining requirements, vendor lock-in, interoperability constraints, uncertainty regarding the generalizability of AI algorithms, and evolving regulatory frameworks. Accordingly, the strongest current case for TLA is in laboratories with sustained specimen volume, stable workflows, and the capacity to maintain robust technical oversight.

Despite rapid progress, AI in microbiology still requires human supervision, particularly for polymicrobial cultures, uncommon morphologies, and the distinction between contamination and true infection (Jacot et al., 2024; Dien Bard et al., 2025). Broader deployment also raises concerns about dataset bias and external validity. Laboratories should therefore ensure that AI tools are trained and evaluated on accurate, representative data and should implement continuous monitoring to detect unexpected performance drift (Cherkaoui et al., 2024; 2025a, b; Windecker et al., 2025). Internal and external quality assessment programs will be essential if these tools are to move from promising local solutions to broadly reliable diagnostic platforms.

## Conclusions

Personnel shortages are accelerating the adoption of robotics and AI in clinical microbiology. Total laboratory automation can improve standardization, throughput, and turnaround time, and AI can extend these gains by supporting digital image analysis, colony recognition, and workflow triage. However, these benefits are not uniform across specimen types, laboratory settings, or diagnostic tasks. This review, grounded in institutional experience and recent literature, highlights both the operational value of automation and the areas in which evidence remains incomplete, particularly regarding AI generalizability, performance benchmarking, and integration with molecular workflows. Phenotypic and molecular methods should be deployed as complementary tools within clearly defined diagnostic pathways. Future progress will depend less on adding technology than on integrating it intelligently through robust information systems, careful validation, and molecular diagnostic stewardship.
